# Clinical Outcomes of Gamma Knife Radiosurgery in the Treatment of Patients with Trigeminal Neuralgia

**DOI:** 10.1155/2012/919186

**Published:** 2011-10-25

**Authors:** Ameer L. Elaimy, Peter W. Hanson, Wayne T. Lamoreaux, Alexander R. Mackay, John J. Demakas, Robert K. Fairbanks, Barton S. Cooke, Sudheer R. Thumma, Christopher M. Lee

**Affiliations:** ^1^Gamma Knife of Spokane, 910 W 5th Avenue, Suite 102, Spokane, WA 99204, USA; ^2^Cancer Care Northwest, 910 W 5th Avenue, Suite 102, Spokane, WA 99204, USA; ^3^MacKay & Meyer MDs, 711 S Cowley Street, Suite 210, Spokane, WA 99202, USA; ^4^Spokane Brain & Spine, 801 W 5th Avenue, Suite 210, Spokane, WA 99204, USA

## Abstract

Since its introduction by Leksell, Gamma Knife radiosurgery (GKRS) has become increasingly popular as a management approach for patients diagnosed with trigeminal neuralgia (TN). For this reason, we performed a modern review of the literature analyzing the efficacy of GKRS in the treatment of patients who suffer from TN. For patients with medically refractory forms of the condition, GKRS has proven to be an effective initial and repeat treatment option. Cumulative research suggests that patients treated a single time with GKRS exhibit similar levels of facial pain control when compared to patients treated multiple times with GKRS. However, patients treated on multiple occasions with GKRS are more likely to experience facial numbness and other facial sensory changes when compared to patients treated once with GKRS. Although numerous articles have reported MVD to be superior to GKRS in achieving facial pain relief, the findings of these comparison studies are weakened by the vast differences in patient age and comorbidities between the two studied groups and cannot be considered conclusive. Questions remain regarding optimal GKRS dosing and targeting strategies, which warrants further investigation into this controversial matter.

## 1. Introduction

Trigeminal neuralgia (TN) is a disorder of cranial nerve (CN) V that results in severe episodes of shock-like or lancinating pain in one or more of its three divisions (V1–V3). TN can be classified into two categories based on etiology: classical and symptomatic [1]. Idiopathic TN and cases due to vascular compression of CN V are categorized as classical TN [1]. Patients diagnosed with symptomatic TN experience trigeminal-related facial pain secondary to a brain tumor, skull deformity, or multiple sclerosis (MS) [1]. Evidence suggests that the majority of cases of TN are the consequence of focal compression of the entry zone of the root of the trigeminal nerve [2], while only 2% of cases are observed in patients diagnosed with MS [3]. Other than excruciating facial pain, there are no other direct medical symptoms associated with TN, and the condition does not decrease life expectancy. However, many patients with TN struggle with accomplishing tasks that affect quality of life, which is how this disorder elicits a negative impact on the social and mental wellness of the patients who suffer from this illness. 

Following the diagnosis of TN, pharmacotherapy is often the initial management approach in achieving facial pain control. However, many patients experience only limited relief from medication or are unable to endure the side effects of the prescribed drugs, and in turn seek neurosurgical intervention. Currently, surgical approaches include microvascular decompression (MVD) or a number of techniques that target the trigeminal ganglion or root which involve the destruction or blockage of portions of those anatomical structures [1, 2]. Although the neurosurgical modalities are preferred in many clinical situations and have proven to be effective in achieving initial pain control, they are known to come with a variety of complications, and facial pain recurrence is likely [4]. 

Stereotactic radiosurgery (SRS) has proven to be an effective management approach for patients with medically [5] and surgically [6] refractory TN as a primary and repeat treatment modality. The use of radiosurgery in the treatment of TN dates back to Sweden in the 1950's, where Professor Lars Leksell performed radiogangliotomies directed at the gasserian ganglion [7]. Since the time of Leksell, advancements in radiosurgery and imaging technologies has led to the increasing popularity of SRS as a treatment option for patients with TN. One form of SRS that can be delivered to a patient is through a machine called the Gamma Knife (GK). The GK device is a cobalt-60-based machine, with 201 separate 4 to 18 mm collimator openings, that emits multiple gamma rays that converge on a target specified by computer planning. For specific medication intolerable patient subsets, Gamma Knife radiosurgery (GKRS) can be used as an initial management approach, or as a secondary management approach following radiosurgery or one or more of the various surgical modalities. As the evidence examining the role of GKRS in the management of patients with TN is increasing, it is of utmost importance for physicians to understand the criteria associated with GKRS, so that the optimal course of treatment for their patients can be prescribed. 

An evidence-based review on the evaluation and treatment of TN by Gronseth et al. [8] found Level C evidence indicating that gasserian ganglion percutaneous techniques, GKRS, and MVD may be considered for facial pain management for medically refractory patients. However, questions remain regarding optimal treatment modalities in specific patient subsets. For this reason, the goal of this paper is to provide a modern review of the literature thoroughly analyzing the efficacy of GKRS in the treatment of patients with TN, as well as evaluating the treatment planning and methods associated with this evolving modality.

## 2. Review of Gamma Knife Radiosurgery for Trigeminal Neuralgia

### 2.1. Literature Search Strategy

To identify contemporary studies assessing the clinical outcomes of patients treated with GKRS for TN, a PubMed search from 2006 to April 2011 was performed. Keywords for search included “Gamma Knife OR Gamma Knife radiosurgery OR stereotactic radiosurgery trigeminal neuralgia OR *tic douloureux*.” Studies analyzed in this review included retrospective cohort studies and prospective cohort studies with ≥5 evaluated patients. Studies published only in abstract form and studies published in a language other than English were excluded from our analysis. Due to our broad search strategy and the vast amount of world literature, references from existing review articles were also selected and analyzed for study inclusion eligibility.

### 2.2. Clinical Outcomes of Patients Undergoing a Single Gamma Knife Treatment

We reviewed a total of 19 studies [4, 5, 9–25] analyzing the efficacy of patients with TN who were treated once with GKRS (Table 1). Thirteen of the 19 evaluated studies [4, 5, 9–19] utilized the Barrow Neurological Institute (BNI) pain intensity scale [26] as a measurement of response to treatment (See Section 3). One of the studies [18] included patients diagnosed with atypical TN. Of these 13 studies, only two [9, 13] analyzed patients treated with GKRS as an initial management approach. With a median followup of 31 months, Sheehan et al. [9] classified 87% of patients in BNI class I-IIIb, while Chen et al. [13] classified 91% of patients in BNI class I-IIIb (median followup = 15 months). Chen et al. [13] also reported that five of the 44 patients (11%) treated with GKRS developed hypoesthesia following the procedure. 

The other 11 BNI pain intensity scale studies we reviewed included patients where previous surgical procedures were performed in a fraction of patients [4, 5, 10–12, 15–19] or all patients [14]. Of the 10 studies where previous surgical procedures were performed in a fraction of patients, nine reported outcomes in terms of categorizing patients in BNI class I-IIIb [4, 5, 10–12, 15–18]. Specifically, Riesenburger et al. [10] classified 58.6% of patients in BNI class I-IIIb (median followup = 48 months), Kondziolka et al. [5] classified 71% of patients in BNI class I-IIIb at three years, Dhople et al. [18] classified 72% of patients in BNI class I-IIIb (median followup = 29 months), Han et al. [11] classified 76.7% of patients in BNI class I-IIIb (mean followup = 58 months), Dhople et al. [17] classified 81% of patients in BNI class I-IIIb (median followup = 5.6 years), Matsuda et al. [16] classified 82% of patients in BNI class I-IIIb (median followup = 37 months), Little et al. [15] classified 83% of patients in BNI class I-IIIb (median followup = 6.3 years), Dellaretti et al. [4] classified 89.5% of patients in BNI class I-IIIb (mean followup = 20.3 months), and Park and Hwang [12] classified 94% of patients in BNI class I-IIIb with a minimum followup of 3 years. Pan et al. [19] reported clinical outcomes with respect to BNI class I, which contained only 5.7% of patients. The study that evaluated GKRS where previous surgical procedures were performed in 100% of patients classified 85% of patients in BNI class I-IIIb, with a median followup of 36 months [14].

We also reviewed two studies that used the excellent-good-fair-poor (EGFP) categorical scale to assess patient outcomes [20, 21] (See Section 3 **)**. Azar et al. [21] treated 30 patients with TN with GKRS at Iran Gamma Knife Center between 2006 and 2007. The authors reported that 40% of patients had an excellent outcome, 10% of patients had a good outcome, 33% of patients had a fair outcome, and 17% of patients had a poor outcome following the procedure. Approximately 13% of patients reported facial numbness related to GKRS. Sekula et al. [20] analyzed 29 consecutive patients who underwent MVD after failed GKRS. After surgery, 15 patients (54%) reported an excellent outcome, one patient (4%) reported a good outcome, two patients (7%) reported a fair outcome, and 10 patients (36%) reported a poor outcome. The complications from MVD included facial numbness in six patients (21%), dysesthesias in three patients (11%), and delayed facial palsy in one patient (4%). 

 Four of the 19 studies we evaluated [22–25] used other methodologies in determining the effectiveness of GKRS. In a prospective controlled trial, Régis et al. [22] analyzed 100 patients with TN treated with GKRS and reported that 83 patients (83%) were completely pain free, 58 of which (58%) discontinued all medication following the procedure (minimum followup = 12 months). Ten patients (10%) experienced radiation-induced complications, which included facial paresthesia or hypesthesia. Knafo et al. [23] performed a study investigating the short-term efficacy of GKRS in 67 patients with medically refractory TN. The authors performed followup assessments at 2, 4, and 6 months. Overall, 77.6% of patients witnessed some degree of pain relief, with 32.6% of those patients becoming completely pain free. Of the 67 patients, 10 (14.9%) experienced complications from the procedure, which included hypoesthesia and paresthesia. Longhi et al. [24] treated 160 patients with TN with GKRS (mean followup = 37.4 months). Sixty eight patients (42.5%) underwent prior invasive treatments. In clinical analysis, it was found that 61% of patients were pain free without medication, 29% of patients were pain free with medication, and 10% of patients did not respond to GKRS. The observed side effects were paresthesia (6.25%) and hypoesthesia (2.5%). Kang et al. [25] treated 77 patients with idiopathic TN with GKRS. Thirty eight patients (49.4%) exhibited some level of pain improvement following GK treatment, with 23 of those patients (29.9%) reporting a pain-free outcome. Twelve patients (15.6%) experienced complications, which were reported to be mild facial sensory changes and mild facial nerve dysfunction.

### 2.3. Clinical Outcomes of Patients Undergoing Multiple Gamma Knife Treatments

As GKRS has proven to be an effective initial treatment for TN, numerous reports have been published analyzing patients treated on multiple occasions (>1) with GKRS. We reviewed six studies evaluating patients treated more than once with GKRS [27–32] (Table 2). Of these six articles, two [29, 32] utilized the BNI pain intensity scale [26]. Gellner et al. [32] evaluated 21 patients treated on two occasions with GKRS. Ten patients (48%) had undergone previous surgical procedures. Sixteen patients (76.2%) exhibited compelling improvements and were placed in BNI class I-II. Huang et al. [29] analyzed 65 medically refractory patients with TN who were treated with GKRS as a second treatment modality. Of these 65 patients, 30 (46%) had undergone GKRS as an initial management approach. The authors placed 22 patients (34%) in BNI class I, 11 patients (17%) in BNI class II, four patients (6%) in BNI class IIIa, and five patients (8%) in BNI class IIIb. Overall, with a median followup of 64 months, 65% of patients reported successful results in terms of pain control rates.

 A total of three of the six reviewed studies evaluated patients using the EGFP categorical scale [28, 30, 31]. Aubuchon et al. [31] analyzed 37 patients treated a second time with GKRS for recurrent TN and reported that 17 patients (46%) achieved excellent pain relief, nine patients (24%) achieved good pain relief, five patients (14%) achieved fair pain relief, and six patients (16%) achieved poor pain relief. However, the authors concluded that 57% of patients experienced some form of trigeminal dysfunction following repeat radiosurgery. Similar to the results reported by Aubuchon et al. [31], Huang et al. [28] treated 28 patients with repeat GKRS and reported that 12 patients (43%) exhibited excellent pain relief, five patients (18%) exhibited good pain relief, and two patients (7%) exhibited fair pain relief. In addition, the authors found a statistically significant (*P* = 0.047) correlation between cumulative radiation doses >115 Gy and facial numbness. In a separate study, Huang et al. [30] evaluated the efficacy of MVD following failed repeat GKRS. Specifically, a total of eight patients underwent MVD a mean of 7.6 months following repeat GKRS. Of the eight patients, seven (87.5%) were completely pain free at a mean of 21 months following neurosurgery. This data supports the use of MVD if multiple GK procedures are deemed ineffective. 

Kimball et al. [27] treated 53 patients with repeat GKRS and analyzed the patients not lost during followup using the Marseille scale [22], which categorizes patients into one of five classes, with a higher class statistically indicating a worse prognosis for the patient. With a mean followup of 42 months, 20 patients (43.5%) were categorized in Marseille class I-II, six patients (13%) were categorized in Marseille class III-IV, and 20 patients (43.5%) were categorized in Marseille class V. The authors also reported a statistically significant (*P* = 0.047) correlation between facial numbness and superior long-term pain relief. A total of 22 patients (48%) experienced trigeminal dysfunction of any kind, while 21 patients (46%) experienced numbness in the face.

### 2.4. Clinical Outcomes of Patients Undergoing Single versus Multiple Gamma Knife Treatments

Since GKRS can be performed as both initial and salvage treatment options for patients who suffer from TN, its efficacy has been compared in patients who undergo one versus multiple radiosurgery procedures. We reviewed eight studies to further examine this matter [3, 33–39] (Table 3). Four of the eight studies utilized the BNI pain intensity scale [26] to evaluate patient outcomes [3, 33–35]. Verheul et al. [33] performed 450 GK procedures in 365 patients. With a median followup of 28 months, it was reported that 75%, 60%, and 58% of patients with idiopathic TN had BNI scores of I–IIIb at 1, 3, and 5 years, respectively. The 1-, 3-, and 5-year-BNI scores of I–IIIb in patients with MS-related TN were 56%, 30%, and 20%, respectively. The authors concluded that repeat GKRS exhibited similar success rates when compared to the initial procedure. Similar to Verheul et al. [33], Park et al. [34] did not find differences in terms of time to initial response, time to pain recurrence, and overall pain relief when comparing patients who underwent one versus two GK treatments. However, it was observed that patients who received two GK treatments were more likely to have facial sensory changes when compared to patients treated a single time with radiosurgery. Little et al. [35] performed a study where 79 patients with typical TN were treated with GKRS as a salvage procedure. Twenty-one patients (27%) underwent GKRS as an initial modality. Approximately five years following salvage GKRS, the authors reported that 50% of patients experienced pain relief and 20% of those patients were completely pain free. In addition, a statistically significant (*P* = 0.029) correlation between GKRS failure and prior MVD was found. Zorro et al. [3] treated 37 patients (78% had failed prior surgery) with MS-related TN with GKRS. Nine patients (24%) underwent GKRS as their first procedure. The reported 1, 3, and 5 year BNI scores of I–IIIb were 82.6%, 73.9%, and 54%, respectively. 

The other four studies we reviewed utilized the EGFP categorical scale as a measurement of response to treatment [36–39]. Two of the evaluated studies [36, 37] were conducted by Fountas et al. and analyzed patients treated with GKRS for idiopathic TN based on whether or not they had undergone previous surgical or radiosurgical procedures for facial pain control. One of the studies evaluated 106 patients (19 previous radiosurgery procedures) and concluded that the treatment group without a previous history of surgical or radiosurgical procedures exhibited superior clinical outcomes, with 1-year and 2-year complete pain relief rates of 82.5% and 78%, respectively [36]. The 1-year and 2-year complete pain relief rate in the patient group with a history of surgical or radiosurgical procedures was 69.4% and 63.5%, respectively [36]. As expected, similar results were found in the other study by Fountas et al. [37]; however, no prior radiosurgical procedures were performed in the patient group with a history of prior procedures. Huang et al. [38] conducted a study where 89 patients with idiopathic TN were treated with GKRS as an initial management approach, 20 of which underwent a subsequent GKRS procedure for facial pain recurrence. Following the initial radiosurgical procedure, 50 patients (56%) had an excellent response, 12 patients (13.5%) had a good response, and 7 patients (7.9%) had a fair response. Following the second radiosurgical procedure, 11 patients (55%) had an excellent response and one patient (5%) had a good response. In a separate study, Huang et al. [39] assessed 21 patients with benign tumor-related TN who were treated with GKRS as an initial or repeat procedure. Following the initial GK procedure to the tumor, 12 patients (57%) had an excellent response and 1 patient (5%) had a good response. A total of eight patients were treated with a subsequent GKRS procedure targeted at the ipsilateral trigeminal root or ganglion due to facial pain recurrence. Following the second radiosurgical procedure, the authors reported four patients (50%) with an excellent response.

### 2.5. Comparison Studies

We identified six studies comparing patients treated with GKRS with patients treated with one of the various surgical modalities [2, 40–44] (Table 4). The authors of this review acknowledge the importance of percutaneous techniques in the management of TN; however, our modern literature search predominantly yielded comparison studies analyzing the efficacy of MVD when compared to GKRS. Specifically, four of the six studies [2, 40–42] analyzed patients treated with GKRS against patients treated with MVD. Linskey et al. [2] prospectively evaluated a total of 80 patients with typical TN. No previous procedures were performed on the patients constituting this study. Specifically, 36 were treated with MVD (45%), while 44 were treated with GKRS (55%). The MVD treatment arm statistically differed from the GKRS treatment arm with respect to age (median of 54 versus 74 years), preoperative symptom duration (median of 2.6 versus 7.5 years), and the presence of major comorbidities (2.8 versus 58.3%). The mean followup time was determined to be 3.4 ± 2.1 years. The authors reported that patients treated with MVD exhibited superior levels of initial (100%), 2 year (88%), and 5 year (80%) actuarial pain-free rates when compared to the patients treated with GKRS (78, 50, and 33%, resp.), with a *P* value of 0.0002. In addition to increased levels of patient satisfaction, as reported by required patient surveys, the MVD treatment group also had a decreased level of permanent mild (5.6%) and severe sensory loss (0%) when compared to the GKRS treatment group (6.8% and 2.3%, resp.). Two patients (5.6%) in the MVD group experienced permanent mild paresthesias or numbness, one (2.8%) patient experienced a cerebrospinal fluid leak from the wound, and one patient (2.8%) experienced hearing loss and diplopia. Three patients (6.8%) in the GKRS group experienced permanent mild paresthesias or numbness, one patient (2.3%) experienced a more permanent sensory numbness, and one patient (2.3%) experienced a transient headache and nausea following the GK procedure. 

Brisman [40] compared 24 patients treated with MVD with 61 patients treated with GKRS. All patients were diagnosed with typical TN and did not undergo previous GK or MVD procedures. It was reported that patients treated with MVD exhibited superior levels of complete pain relief at 12 (68%) and 18 months (68%) when compared to the GKRS group, who's complete pain relief rate was 58% at 12 months and 24% at 18 months (*P* = 0.089). The treatment arms did not statistically differ in terms of ≥90% pain relief at 12 and 18 months. No permanent complications were observed. This study could be criticized due to the large difference in the number of patients constituting the two treatment arms. 

Oh et al. [41] evaluated a total of 45 elderly patients (>65 years of age) diagnosed with idiopathic TN who were treated with either MVD (27 patients) or GKRS (18 patients). It was reported that three MVD patients (11%) and three GK patients (17%) underwent previous percutaneous procedures. The mean followup period was reported to be 35.9 months for the MVD group and 33.1 months for the GKRS group. According to the BNI pain intensity scale [26], the MVD group had a superior prognosis, with 17 patients (63%) classified in BNI class I-II compared with the 10 patients (56%) in the GKRS group classified in BNI class I-II. The two groups did not differ in terms of pain recurrence during followup. The observed complications following MVD included constant headache in 11 patients (40.7%), facial paresthesia in five patients (18.5%), paresthesia of the tongue in two patients (7.4%), infection at the site of incision in one patient (3.7%), an acute subdural hemorrhage in one patient (3.7%), temporary hearing loss in one patient (3.7%), and otitis media with cerebrospinal leakage in one patient (3.7%). Two patients (11%) in the GKRS group experienced paresthesia. 

Aryan et al. [42] compared the clinical outcomes of 19 patients treated with MVD with 15 patients treated with GKRS. Patients diagnosed with symptomatic TN were excluded from this study. Nine GK patients (60%) and four MVD patients (21%) underwent previous surgical procedures. The treatment arms statistically differed (*P* = 0.0005) with respect to mean patient age, with the mean age of the GKRS group exceeding the MVD group by 13 years (74 versus 61 years). The median followup was determined to be 17 months. The authors determined clinical results by using the EGFP categorical scale. In addition, patient satisfaction was graded on a scale of 1 (unsatisfied) to 10 (completely satisfied). It was reported that the mean TN complexity grade was statistically different (*P* < 0.001) between the treatment arms (GK = 5.8; MVD = 3). The average response following the procedure was determined to be 3.4 for the MVD group and 2.4 for the GKRS group (*P* = 0.017). Also, it was found that the satisfaction score for the MVD group was superior to the GKRS group (8.7 versus 6.4), with a *P* value of 0.02. The authors reported a statistically significant correlation between TN complexity grade and clinical response (*P* < 0.001), as well as TN complexity grade and patient satisfaction (*P* < 0.001). 

To date, no randomized trials have been conducted analyzing the outcomes of patients with TN who are treated with MVD compared to GKRS. In a large review on TN management, Zakrzewska and Linskey [45] found evidence that MVD is an effective treatment for long-term facial pain relief but comes with an increased risk of ipsilateral hearing loss. In addition, the authors concluded that single-dose SRS is an effective treatment for long-term facial pain relief but puts patients at risk for facial numbness or facial paresthesias. Investigation into this matter in the form of a randomized controlled trial would provide the best evidence in terms of facial pain relief and procedure-related complications. 

In addition, we reviewed two studies comparing patients treated with GKRS with patients treated with posterior fossa exploration (PFE) [43, 44], both of which were conducted by Pollock and colleagues at the Mayo Clinic College of Medicine. One of the studies [43] was a specific prospective comparison of 91 patients treated with PFE and 49 patients treated with GKRS for idiopathic TN as an initial management approach. The treatment arms statistically differed in terms of age (GKRS = 67.1 years; PFE = 58.2 years), with a *P* value <0.001. The median followup time was 38 months. It was reported that patients treated with PFE were more likely to be pain free and off medications at 1 year (84%) and 4 years (77%) when compared to the GKRS group (66 and 56%, resp.) (*P* = 0.003). Retreatment for recurrent facial pain was performed in 15% of the patients in the PFE treatment arm and 35% of patients in the GKRS treatment arm (*P* = 0.009). Also, it was found that nonbothersome facial numbness occurred more frequently in the GKRS group (*P* = 0.04). An additional study from the Mayo Clinic evaluated patients with recurrent TN who underwent 3 or more surgical procedures [44]. The authors reported that patients treated with PFE exhibited superior levels of complete pain relief at 3 years of followup when compared to patients treated with GKRS, balloon compression, and glycerol rhizotomy (*P* < 0.01) and underwent additional surgery for recurrent facial pain less often when compared to patients treated with the other modalities (*P* = 0.02). Clinical 006Futcomes did not differ between patients treated with GKRS and patients treated with the percutaneous techniques.

## 3. Treatment Planning and Methods

### 3.1. Types of Radiosurgery

SRS can be performed by a variety of tools, which include GKRS, CyberKnife technology, and linear accelerator (LINAC)-based treatment. Our analysis yielded one study whose primary endpoint was to devise a method using CyberKnife treatment planning that would mimic the dosimetric characteristics of the GK treatment plan in five patients undergoing radiosurgery for TN [46]. The position of the trigeminal nerve was determined using computed tomography cisternography. Both the isodose lines and critical structures were identified using the GKRS treatment plan and were transferred to the CyberKnife treatment planning system. It was reported that the average length of the trigeminal nerve receiving a dose of 60 Gy was 4.5 mm for the GK, 4.5 mm for the nonisocentric CyberKnife, and 4.4 mm for the isocentric CyberKnife. The authors found it more difficult to minimize the dose to critical structures when using CyberKnife technology. Also, the dose falloff of GKRS was found to be steeper when compared to CyberKnife technology due to, what the authors hypothesized, the large number of gamma rays produced which converge on the focal point with precision. 

As previously mentioned, the GK machine's primary functional unit is cobalt-60, which is used to emit photon energy through 201 separate 4 to 18 mm collimator openings that converge on a target specified by a treatment planning system. Balamucki et al. [47] performed a study examining if the half life of cobalt (5.26 years) relates to the outcomes for patients being treated for TN with GKRS. The authors collected data on 239 GKRS procedures performed at their institution between 1999 and 2004. Patient surveys were used to measure responses to radiosurgery. With the followup time ranging from one to six months, it was reported that 80% of patients experienced some degree of pain relief and that 56% of those patients were pain free. The authors concluded that clinical outcomes remained consistent during the first half life of cobalt-60.

### 3.2. Dosing

An area of controversy in the treatment of patients with TN is defining the optimal maximum radiosurgery dose that can be delivered to specific patient subsets. We analyzed five studies whose primary endpoint was to assess GKRS-dosing efficacy [48–52]. Kim et al. [48] utilized the BNI pain intensity scale [26] to assess 66 TN patients treated with a GK maximum dose of 80 Gy and 44 TN patients treated with a GK dose of 85 Gy. Although the two groups did not statistically differ in terms of facial rain relief and procedure-related complications, the authors did report that patients treated with a GK dose of 85 Gy experienced a more rapid response to treatment when compared to the patients treated with a GK dose of 80 Gy. Arai et al. [50] analyzed 165 patients with TN treated with a GKRS dose of 80 Gy. Specifically, the authors divided the patients into two groups, which differed in the radiation dose rate received (low-dose rate = 1.21–2.05 Gy/min; high-dose rate = 2.06–3.74 Gy/min). Using the BNI pain intensity scale [26] as a clinical evaluation method, it was reported that the low-dose-rate group and the high-dose-rate group did not statistically differ in terms of initial pain relief, maintenance of pain relief, and clinical complications. 

Massager et al. [49] divided 358 patients with TN into three treatment groups. Patients in group one were treated with a GK dose <90 Gy with no beam channel plugging, patients in group two were treated with a GK dose equal to 90 Gy with no beam channel plugging, and patients in group three were treated with a GK dose equal to 90 Gy with beam channel plugging. Although the trend did not reach full statistical significance (*P* = 0.054), patients in group three exhibited the highest level of pain relief, while patients in group one exhibited the lowest level of pain relief. The authors also observed that the three groups statistically differed (*P* < 0.0001) in terms of trigeminal nerve dysfunction, with patients in group three experiencing the highest rate of mild and bothersome complications and patients in group one experiencing the lowest rate of mild and bothersome complications. Similar to the results of Massager et al. [49], Morbidini-Gaffney et al. [52] reported positive outcomes in patients treated with GK doses >85 Gy. The authors also found that patients treated with two isocenters were more likely to have superior BNI pain intensity scale [26] scores during their course of followup when compared to patients treated with a single isocenter. 

Dvorak et al. [51] analyzed GKRS retreatment doses in 28 patients. The median initial dose was 80 Gy, and the median retreatment dose was 45 Gy. Although the authors did not report any predictors in terms of facial pain control and patient morbidity, they did compare the results of their study with seven published retreatment articles and found that successful levels of pain control (>50%) were significantly correlated with cumulative GKRS doses >130 Gy, as well as new trigeminal nerve dysfunction (>20%).

### 3.3. Targeting

In addition to dose selection efficacy in select patient cohorts, the radiosurgical target of CN V is another subject matter that requires further clinical investigation. We reviewed three studies [53–55] analyzing specific GKRS targeting methods in the treatment of TN and one study [56] that examined the accuracy of GKRS to its image-guided target. Matsuda et al. [53] compared patients treated with GKRS targeted at the dorsal root entry zone (59 patients) with patients whose radiosurgical target was the retrogasserian zone of the trigeminal nerve (41 patients). With a median followup of 30 months, the dorsal root entry target group exhibited statistically superior levels of initial complete pain remission (*P* = 0.003) and experienced less complications than the retrogasserian zone group (*P* = 0.009). Chen et al. [54] also reported positive results with the dorsal root entry zone-targeting approach, with a success rate of 82.8% and a complication rate of 15%. Park et al. [55] compared the dorsal root entry zone and retrogasserian zone-targeting methods in the treatment of 39 patients with medically refractory TN. The authors reported that the two treatment arms did not statistically differ in treatment success (BNI class I-IIIb) with respect to the BNI pain intensity scale [26]. However, patients treated with the retrogasserian zone-targeting method experienced a substantially shorter time of response to GKRS than patients treated with the dorsal root entry zone-targeting method (*P* = 0.044). Although the two groups did not statistically differ with regard to treatment-related morbidities, it was found that the patients whose targeting approach was the dorsal root entry zone experienced a greater amount of bothersome complications than the retrogasserian zone group. 

Massager et al. [56] analyzed the targeting accuracy of GKRS in 65 patients treated for TN whose six month followup MRI showed focal contrast enhancement of the trigeminal nerve. The authors found that the median deviation of the coordinates between the intended radiosurgical target and the center of contrast enhancement was 0.91 mm in Euclidean space. The median radiation dose fitting into the contrast enhancement region was determined to be 77 ± 8.7 Gy. This small deviation from the GKRS target explains the high accuracy and precise nature of the machine.

### 3.4. Measurements of Response to Treatment

The two most common methods of measuring patient outcomes from GKRS in the management of TN are the Barrow Neurological Institute pain intensity scale [26] (Table 5) and the excellent-good-fair-poor (EGFP) categorical scale (Table 6). The BNI pain intensity scale divides patients into one of five classes, with a higher class indicating a worse prognosis for the patient. Patients in BNI class I experience no trigeminal pain and do not require medication. Patients in BNI class II experience occasional trigeminal pain but do not require medication. Patients in BNI class IIIa do not experience trigeminal pain but require the use of medication. Patients in BNI class IIIb experience some trigeminal pain that can be satisfactorily managed with medication. Patients in BNI class IV experience some trigeminal pain that is not satisfactorily managed with medication. Patients in BNI class V do not experience a reduction in pain. The EGFP method categorizes patients into one of four groups. “Excellent” outcomes are defined as complete pain relief without the need of medication. “Good” outcomes are defined as complete pain relief with the need of medication. “Fair” outcomes are defined as a >50% pain relief rate. “Poor” outcomes are defined as a <50% pain relief rate or treatment failure.

## 4. Conclusions

For patients with medically refractory forms of TN, GKRS has proven to be an effective initial and repeat treatment option. Cumulative research suggests that patients treated a single time with GKRS exhibit similar levels of facial pain control when compared to patients treated multiple times with GKRS. However, patients treated on multiple occasions with GKRS are more likely to experience facial numbness and other facial sensory changes when compared to patients treated once with GKRS. Although numerous articles have reported MVD to be superior to GKRS in achieving facial pain relief, the findings of these comparison studies are weakened by the vast differences in patient age and comorbidities between the two studied groups and cannot be considered conclusive. Further evidence in the form of a Phase III-randomized trial is needed to confirm the clinical outcomes of patients treated with either modality. Questions remain regarding optimal GKRS dosing and targeting strategies, which warrants further investigation into this controversial matter.

## Figures and Tables

**Table 1:** Clinical outcomes of patients undergoing a single Gamma Knife treatment.

Author (year)	Clinical evaluation method	GKRS max dose (Gy)	Study endpoints	Results
			Patients with vessel impingement	59%
			Pain relief in patients with or w/o vascular impingement	*P *= NS
			BNI score I	57%
Sheehan et al. [9] (2010)	BNI	Median: 84	BNI score II	17%
			BNI score III	13%
			BNI score IV	10%
			BNI score V	2%

			BNI score I	43%
Chen et al. [13] (2010)	BNI	90	BNI score I–IIIb	91%
			Patients with hypoesthesia	11%

			BNI score I	32.1%
			BNI score II	3.8%
Riesenburger et al. [10] (2010)	BNI	Median: 80	BNI score IIIa	1.9%
			BNI score IIIb	20.8%
			BNI score IV	41.5%
			Patients with facial numbness	36%

			1-y BNI score I–IIIb	80%
			3-y BNI score I–IIIb	71%
Kondziolka et al. [5] (2010)	BNI	60–90	5-y BNI score I–IIIb	46%
			10-y BNI score I–IIIb	30%
			Patients with facial numbness or paresthesia	10.5%

			BNI score I	22%
Dhople et al. [18] (2007)	BNI	Median: 75	BNI score II	6%
			BNI score III	44%
			Patients with trigeminal dysfunction	19%

			BNI score I–IIIb	76.7%
Han et al. [11] (2009)	BNI	Mean: 79.7	Patients with pain recurrence	52.2%
			Patients with radiation-induced cranial neuropathy	15%

			1-y actuarial rate of freedom from treatment failure	60%
			3-y actuarial rate of freedom from treatment failure	41%
Dhople et al. [17] (2009)	BNI	Median: 75	5-y actuarial rate of freedom from treatment failure	34%
			7-y actuarial rate of freedom from treatment failure	22%
			Superior response duration in patients w/o prior surgery	*P* < 0.02
			Patients with facial numbness	6%

			BNI score I-IIIb	82%
Matsuda et al. [16] (2010)	BNI	80–90	Patients with trigeminal nerve dysfunction	41.3%

			7-y GKRS initial treatment pain-free rate	45%
Little et al. [15] (2008)	BNI	70–90	7-y GKRS secondary treatment pain-free rate	10%
			Patients with bothersome facial numbness	5%

			1-y complete pain relief rate	83.1%
			2-y complete pain relief rate	70.9%
Dellaretti et al. [4] (2008)	BNI	Mean: 85.1	3-y complete pain relief rate	62.5%
			Superior pain relief in patients w/o prior surgery	*P* < 0.05
			Patients with trigeminal dysfunction	21%

			BNI score I	17.6%
			BNI score II	17.6%
Park and Hwang [12] (2011)	BNI	80–90	BNI score IIIa	41.2%
			BNI score IIIb	17.6%
			BNI score V	5.9%
			Patients with trigeminal nerve dysfunction	23.5%

			BNI score I	5.7%
Pan et al. [19] (2010)	BNI	80	Patients with pain recurrence	44.2%
			Patients with facial numbness	9.6%

			1-y BNI score I	26%
Kano et al. [14] (2010)	BNI	60–90	1-y BNI score 1-IIIb	85%
			Patients with trigeminal sensory loss or paresthesia	9.3%

			Excellent outcome	40%
			Good outcome	10%
Azar et al. [21] (2009)	EGFP	90	Fair outcome	33%
			Poor outcome	17%
			Patients with facial numbness	13%

			Excellent outcome	54%
			Good outcome	4%
Sekula et al.* [20] (2010)	EGFP	NR	Fair outcome	7%
			Poor outcome	36%
			Patients with facial numbness	21%
			Patients with dysesthesias	11%

			Patients with complete pain relief	83%
Régis et al. [22] (2006)		Median: 85	Patients with facial numbness	6%
			Patients with hypesthesia	4%

			Patients with complete pain relief	32.6%
Knafo et al. [23] (2009)		80	Patients with significant pain relief	77.6%
			Patients with sensory side effects	14.9%

			Patients pain-free w/o medication	61%
Longhi et al. [24] (2007)		75–95	Patients pain-free with medication	29%
			Patients with no pain relief	10%
			Patients with side effects	9%

			Patients with complete pain relief	29.9%
Kang et al. [25] (2008)		Mean: 84.3	Patients with pain improvement	49.4%
			Patients with side effects	15.6%

BNI: Barrow Neurological Institute; EGFP: excellent-good-fair-poor; GKRS: Gamma Knife radiosurgery; MVD: microvascular decompression; NR: not reported; NS: nonsignificant.

*Study includes patients treated with MVD after failed GKRS.

**Table 2:** Clinical outcomes of patients undergoing multiple Gamma Knife treatments.

Author (year)	Clinical evaluation method	GKRS max retreatment dose (Gy)	Study endpoints	Results
			BNI score I	47.6%
Gellner et al. [32] (2008)	BNI	Mean: 74.3	BNI score II	28.6%
			BNI score III	23.8%

			BNI score I	34%
			BNI score II	17%
			BNI score IIIa	6%
Huang et al. [29] (2010)	BNI	Mean: 49	BNI score IIIb	8%
			1-y pain control rate	74%
			2-y pain control rate	71%
			3-y pain control rate	66%
			Facial numbness	17%

			Excellent pain relief	46%
			Good pain relief	24%
Aubuchon et al. [31] (2010)	EGFP	Mean: 84.4	Fair pain relief	14%
			Poor pain relief	16%
			Trigeminal nerve dysfunction	57%

			Excellent pain relief	43%
Huang et al. [28] (2006)	EGFP	Mean: 52	Good pain relief	18%
			Fair pain relief	7%
			Facial numbness	36%

			Marseille class I-II	43.5%
Kimball et al. [27] (2010)	Marseille	70	Marseille class III-IV	13%
			Marseille class V	43.5%
			Facial numbness	46%

BNI: Barrow Neurological Institute; EGFP: excellent-good-fair-poor; GKRS: Gamma Knife radiosurgery.

**Table 3:** Clinical outcomes of patients undergoing single versus multiple Gamma Knife treatments.

Author (year)	Clinical evaluation method	GKRS max dose (Gy)	Study endpoints	Results
			BNI scores of I–IIIb at 1-y for idiopathic TN	75%
			BNI scores of I–IIIb at 3-y for idiopathic TN	60%
			BNI scores of I–IIIb at 5-y for idiopathic TN	58%
Verheul et al. [33] (2010)	BNI	1st treatment: 80 2nd treatment: 80	BNI scores of I–IIIb at 1-y for MS-related TN	56%
			BNI scores of I–IIIb at 3-y for MS-related TN	30%
			BNI scores of I–IIIb at 5-y for MS-related TN	20%
			5-y idiopathic retreatment pain relief rate	75%
			5-y MS retreatment pain relief rate	46%

			Pain outcome in primary versus secondary GKRS	*P* = NS
Park et al. [34] (2011)	BNI	1st treatment mean: 82.4 ± 6.25 2nd treatment mean: 81 ± 4.89	Primary GKRS facial numbness	21%
			Secondary GKRS facial numbness	45.8%

			BNI score of I–III at 1-y	75%
			Patients requiring additional surgery	41%
Little et al. [35] (2009)	BNI	1st treatment: 80 2nd treatment: 40–50	Patients with mild facial numbness	76%
			Patients with bothersome facial numbness	8%
			Patients with eye symptoms	12%

			BNI scores of I–IIIb at 1-y	82.6%
Zorro et al. [3] (2009)	BNI	Median: 80	BNI scores of I–IIIb at 3-y	73.9%
			BNI scores of I–IIIb at 5-y	54%
			Patients with facial sensory dysfunction	5.4%

			1-y no previous treatment excellent outcome rate	82.5%
			1-y previous treatment excellent outcome rate	69.4%
Fountas et al. [36] (2007)	EGFP	Median: 80	2-y no previous treatment excellent outcome rate	78%
			2-y previous treatment excellent outcome rate	63.5%
			No previous treatment paresthesia rate	15.8%
			Previous treatment paresthesia rate	16.3%

			1-y no previous treatment excellent outcome rate	80.8%
			1-y previous treatment excellent outcome rate	69.2%
Fountas et al. [37] (2006)	EGFP	Median: 80	2-y no previous treatment excellent outcome rate	64%
			2-y previous treatment excellent outcome rate	11.5%
			No previous treatment facial numbness rate	17.3%
			Previous treatment facial numbness rate	16%

			1st treatment excellent outcome rate	56%
			1st treatment good outcome rate	13.5%
Huang et al. [38] (2008)	EGFP	1st treatment mean: 79 2nd treatment mean: 52	1st treatment fair outcome rate	7.9%
			2nd treatment excellent outcome rate	55%
			2nd treatment good outcome rate	5%
			Facial numbness associated with repeat GKRS	*P* = 0.007

Huang et al. [39] (2008)	EGFP	Mean: 60.7	Excellent outcome rate after GKRS to the tumor	57%
			Excellent outcome rate after GKRS to CN V	50%

BNI: Barrow Neurological Institute; CN: cranial nerve; EGFP: excellent-good-fair-poor; GKRS: Gamma Knife radiosurgery; NS: nonsignificant.

**Table 4:** Comparison studies.

Author, surgery type (year)	GKRS max dose (Gy)	Study endpoints	Surgery	GKRS	*P* value
		Initial actuarial pain-free rate	100%	78%	0.0002*
Linskey et al. [2], MVD (2008)	80–90	2-y actuarial pain-free rate	88%	50%	
		5-y actuarial pain-free rate	80%	33%	

		12-mo complete pain relief rate	68%	58%	0.089*
Brisman [40], MVD (2007)	75	18-mo complete pain relief rate	68%	24%	
		Patients requiring retreatment	4.2%	18%	NS

		BNI Grade I-II classification	63%	56%	NR
Oh et al. [41], MVD (2008)	Mean: 77.8	Patients with pain recurrence	11.1%	11.1%	NR

		TN complexity grade	3	5.8	<0.001
Aryan et al. [42], MVD (2006)	90	Average response following treatment	3.4	2.4	0.017
		Patient satisfaction	8.7	6.4	0.02

Pollock and Schoeberl [43], PFE (2010)	Median: 85	1-y pain-free rate	84%	66%	0.003*
		4-y pain-free rate	77%	56%	
		Retreatment rate	15%	35%	0.009

Pollock and Stein** [44], PFE (2010)	Median: 76.1	Patients treated with additional surgery	22%	48%	0.02

BNI: Barrow Neurological Institute; GKRS: Gamma Knife radiosurgery; MVD: microvascular decompression; NR: not reported; NS: nonsignificant; PFE: posterior fossa exploration; TN = trigeminal neuralgia.

*P value indicates overall pain-free levels.

**Data includes patients treated with ≥3 prior operations.

**Table 5:** Barrow Neurological Institute pain intensity scale [26].

*BNI Class I*
No trigeminal pain; no medication
*BNI Class II*
Some trigeminal pain; no medication
*BNI Class IIIa*
No trigeminal pain; managed with medication
*BNI Class IIIb*
Persistent trigeminal pain; managed with medication
*BNI Class IV*
Some trigeminal pain; not adequately managed with medication
*BNI Class V*
Severe pain or treatment failure

**Table 6:** Excellent-good-fair-poor categorical scale.

*Excellent*
Complete pain relief without medication
*Good*
Complete pain relief with medication
*Fair*
>50% pain relief
*Poor*
<50% pain relief
